# Charting the course for new discoveries in polyploid lineages

**DOI:** 10.1002/aps3.11613

**Published:** 2024-08-21

**Authors:** Michael R. McKain, Ya Yang, Agnieszka Golicz, Briana L. Gross

**Affiliations:** ^1^ Department of Biological Sciences University of Alabama 411 Mary Harmon Bryan Hall, 500 Hackberry Lane Tuscaloosa 35487 Alabama USA; ^2^ Department of Plant and Microbial Biology University of Minnesota Twin Cities 1445 Gortner Avenue St. Paul 55108 Minnesota USA; ^3^ Department of Agrobioinformatics, IFZ Research Center for Biosystems, Land Use and Nutrition Justus Liebig University Heinrich Buff Ring 26 Giessen 35392 Germany; ^4^ Department of Biology, University of Minnesota Duluth 207 Swenson Science Building, 1035 Kirby Drive Duluth 55812 Minnesota USA

Methods for generating and analyzing data from polyploid species are not new to *Applications in Plant Sciences*, yet a special issue on the topic still presents an exciting opportunity to explore newly emerging research techniques. The complexity associated with the existence of multiple genomes in a single nucleus has meant that despite decades of research, there are still unexplored frontiers at the molecular, phylogenetic, ecological, and evolutionary levels. Some uncharted areas persist despite the forays of excellent research by dedicated scientists, and some remain unmapped because the community avoids polyploid species due to a lack of tools or data. The eight articles in this special issue provide new waypoints and allow us to push the boundaries of our knowledge of polyploid lineages. The tools and applications offered here range from critical techniques for determining the ploidy level of an organism, through synthetic reviews of the optimal treatment of polyploid data for phylogenomics and population genomics, to leveraging and developing new tools to further our understanding of genome dynamics and whole‐plant responses to polyploidy. We look forward to the impact that these tools and innovative approaches will have in accelerating the expansion of research into the nature and impact of polyploidy across plant taxa in the coming years.

Despite the generally acknowledged prevalence of polyploidy across plants, determining the ploidy of any given species or specimen is far from trivial. Techniques for answering this question include direct chromosome counts, but also indirect measures through flow cytometry (Smith et al., 2018), measurements of spore sizes (Kuo et al., 2021), and even spectroscopy (Buono and Albach, 2023). This issue features two new tools to facilitate the accurate assessment of ploidy—one method with a long tradition in plant science, and another that takes advantage of modern sequencing data. Ramirez‐Castillo et al. (2024) developed a method using croziers, or fiddleheads, to count chromosomes in different fern species. Although roots are typically used for mitotic chromosome counts, the ability to incorporate croziers as potential sources of material allows for a wider array of availability for samples. Ramirez‐Castillo et al. use an enzyme pretreatment with a cellulose–pectinase solution to improve permeability of the tissue for the uptake of colchicine to arrest chromosomes at metaphase. Chromosome counting is the original method by which polyploidy was first described in plants (reviewed in Soltis et al., 2014), and the method of Ramirez‐Castillo et al. continues this legacy. Moving from chromosomes to sequence data, Gaynor et al. (2024) present nQuack, an R package that allows for ploidy estimation from sequence data ranging from whole‐genome resequencing to target enrichment. Building on the methodology of nQuire (Weiß et al., 2018), nQuack implements an expectation maximization with three potential distributions—normal, beta, and beta‐binomial—to identify ploidy level from diploid to hexaploid. This method expands the capacity of polyploidy research by opening up ploidy‐level assessment to any specimen for which DNA can be adequately isolated and sequenced, including herbarium specimens.

The generation of high‐throughput sequencing reads in polyploids has increased the total amount of genetic information available, but researchers face significant challenges in processing that data using tools that are generally conceptualized for diploid organisms. Although the community remains enthusiastic about the potential for unlocking discoveries in polyploids with these genetic approaches, they are rightfully wary of the pitfalls associated with incorrect read mapping and the challenges of correctly identifying homeologs. This issue features two synthetic reviews to guide researchers through the population genomic and phylogenetic exploration of polyploid lineages. Phillips (2024) explores the challenges of genomic variant identification in polyploids and provides detailed recommendations for modifying standard variant calling pipelines to accommodate polyploid data analysis. This paper highlights potential pitfalls researchers will face in this process, including the necessity of accounting for genotype dosage, paralogy between subgenomes, and other polyploid‐specific features that can bias genotype estimation. Ning et al. (2024) review the challenges and limitations of inferring species relationships of polyploids using phylogenomic and transcriptomic approaches. They conclude that, despite improvements in our understanding of species relationships in polyploid‐rich genera, certain challenges remain, including reliable identification of orthologous genes and sorting all homoeologous copies for allopolyploids. Both of these reviews provide critical insights into the foundational analytical approaches required for unraveling the complexities of polyploid genomics, and they provide much‐needed practical recommendations for researchers grappling with polyploid data.

Once allopolyploids have been identified and perhaps placed in a phylogenetic context, it becomes compelling to examine the interaction between the subgenomes. Authors in this special issue took innovative approaches to tackling the question of subgenome assignment and genetic exchange, applying techniques that were originally developed for very different purposes to questions around polyploid genomes. Ortiz and Sharbrough (2024) modified the ABBA‐BABA test (Durand et al., 2011)—developed to identify patterns of interspecific hybridization—in order to assess the patterns of homoeologous gene flow between subgenomes. The modified method tests whether one subgenome has become more like the other or vice versa. To test the method, the authors explored the *Coffea arabica* L. genome, a known allopolyploid between maternal subgenome donor *C. eugenioides* S. Moore and paternal subgenome donor *C. canephora* Pierre ex A. Froehner. Ortiz and Sharbrough were able to identify patterns of homoeologous gene flow associated with both subgenomes being overwritten by the other. Interestingly, the pattern extended to genes associated with plastids being driven to become more maternal‐like, suggesting potential nuclear–plastid incompatibility with the paternal genome. This method allows for a better understanding of homoeologous gene dynamics of subgenomes. Reynolds et al. (2024) take advantage of the similarity between allopolyploid genomes and metagenomics. They used *k*‐mer profiles from the rapidly evolving repetitive elements in genome assemblies to assign polyploid genomes as auto‐ vs. allopolyploids and identify progenitors for the subgenomes. The method has the advantage of being computationally efficient and scalable, and the authors were able to recreate known relationships from well‐studied polyploid lineages using this approach. Both of these new techniques reveal the power of leveraging existing analytical approaches to expand our understanding of polyploid origins and subgenome dynamics.

While the interacting genomes present in allopolyploids can be assigned and understood at the chromosome level, the ultimate expression of these traits is at the organismal level, and this represents its own challenges. How do researchers integrate multiple phenotypic, physiological, and ecological measures to understand the evolution of polyploid organisms beyond their origins and subgenome dynamics? Authors in this special issue offer two new ways of approaching this question, from a careful examination of integrated phenotypic changes within a polyploid lineage to a systematic comparison of hybrid lineages to their progenitors on multiple dimensions. Baker et al. (2024) used methods for gene co‐expression analyses to build networks using anatomical, morphological, and physiological traits. They then compared networks between diploid and allopolyploid species of *Brassica*, identified key traits that affect network structure, and found allopolyploids had larger and more connected networks than diploids. The framework of considering phenotypic traits as networks instead of individual variables is applicable to evolutionary questions that go beyond simple bivariate comparisons, and will help us develop a deeper understanding of the impacts of selection on non‐target traits. The integration of diverse measures associated with polyploids, ranging from niche space to morphology, has historically been challenging. To address this, Krieg (2024) has developed the divergence index (DI), which allows for the investigation and interpretation of patterns associated with hybrids and allopolyploids across diverse types of data and studies. The DI framework allows for novel insights into how a hybrid/allopolyploid differs from its parents in a quantitative manner that works across variables and scales, from phenotypes to niche divergence. This standardized DI approach has the potential to allow a comprehensive assessment of the patterns associated with allopolyploid lineage formation specifically and hybrid lineage formation more generally. Both of these new approaches provide pathways for researchers to fully explore the consequences of hybridization and polyploidy on plant lineages, and we look forward to the new findings that will result from their implementation across taxa.

As noted in the *American Journal of Botany* companion issue (Barker et al., 2024), the range and scope of questions that we can ask and answer in polyploids has vastly increased in the past decade. The papers in this issue set the stage for the next round of empirical innovations, addressing the broad array of challenging, but exciting, ways that researchers can chart new territory and push the boundaries of our knowledge of polyploid lineages, ranging from fundamental questions of ploidy level to complex phenotypic networks. The reviews by Phillips (2024) and Ning et al. (2024) emphasize the impressive progress in the field, synthesizing analytical techniques for processing genomic data from complex polyploid genomes in ways that were not previously possible. At the same time, the new approaches for actually determining the ploidy of individual lineages (Gaynor et al., 2024; Ramirez‐Castillo et al., 2024) are extremely valuable, providing key information without which researchers cannot embark on downstream analyses. Between these two extremes are methods and applications that can further our understanding of the origins and dynamics of subgenomes in polyploid lineages (Ortiz and Sharbrough, 2024; Reynolds et al., 2024), as well as an innovative approach to interrogate phenotypic integration in polyploids (Baker et al., 2024) and a new framework for systematically comparing the phenotypic and ecological divergence of allopolyploids from progenitor lineages (Krieg, 2024). Reflecting on the advances featured here, we hope that they will allow researchers to continue the rapid expansion beyond traditional study systems into new taxa and new geographies.

## AUTHOR CONTRIBUTIONS

This special issue was conceived and developed by the authors. M.R.M., Y.Y., and A.G. carried out all editorial duties for the manuscripts included in this special issue. B.L.G. led the writing and editing of the manuscript, with all the authors contributing text and edits to the final version. All authors approved the final version of the manuscript.
